# Role of RhoC in cancer cell migration

**DOI:** 10.1186/s12935-021-02234-x

**Published:** 2021-10-09

**Authors:** Yingyue Lou, Yuhan Jiang, Zhen Liang, Bingzhang Liu, Tian Li, Duo Zhang

**Affiliations:** 1 Department of Plastic and Reconstructive Surgery, The First Hospital of Jilin University, Changchun, Jilin China; 2 Department of Neurology and Neuroscience Center, The First Hospital of Jilin University, Changchun, China

**Keywords:** RhoC, Cell migration, Three-dimensional, Polarization, Adhesion, Amoeboid

## Abstract

Migration is one of the five major behaviors of cells. Although RhoC—a classic member of the Rho gene family—was first identified in 1985, functional RhoC data have only been widely reported in recent years. Cell migration involves highly complex signaling mechanisms, in which RhoC plays an essential role. Cell migration regulated by RhoC—of which the most well-known function is its role in cancer metastasis—has been widely reported in breast, gastric, colon, bladder, prostate, lung, pancreatic, liver, and other cancers. Our review describes the role of RhoC in various types of cell migration. The classic two-dimensional cell migration cycle constitutes cell polarization, adhesion regulation, cell contraction and tail retraction, most of which are modulated by RhoC. In the three-dimensional cell migration model, amoeboid migration is the most classic and well-studied model. Here, RhoC modulates the formation of membrane vesicles by regulating myosin II, thereby affecting the rate and persistence of amoeba-like migration. To the best of our knowledge, this review is the first to describe the role of RhoC in all cell migration processes. We believe that understanding the detail of RhoC-regulated migration processes will help us better comprehend the mechanism of cancer metastasis. This will contribute to the study of anti-metastatic treatment approaches, aiding in the identification of new intervention targets for therapeutic or genetic transformational purposes.

## Background

Migration is one of the most desirable behavioral attributes of cells, which has been observed in most animal cells and several unicellular organisms, such as amoebae [1]. Cell migration is essential in immune monitoring, wound repair, and embryonic development. Abnormal cell migration is a hallmark of various pathologies such as cancer metastasis and chronic inflammation [1]. Generally, when studying cellular migration behavior, the migration mechanism adopted by cells is classified as either two- (2D) or three-dimensional (3D) migration under in vitro culture and in vivo conditions, respectively [2–4]. According to classic two-dimensional cell migration cycle [5, 6], cell migration is initiated with morphological polarization, in which after cells extend a protrusion in the direction of movement and forms a new cell–matrix adhesion between the protrusion and the cell substrate. The cell body then contracts and moves forward, finally ending the cycle by retracting adhesions at the rear [7]. In recent years, as research has intensified, the essence of cell migration—namely 3D migration that includes three modes: mesoscopic, lobopodial, and amoeboid migration—has surfaced. In 3D migration, the cell interacts with the surrounding matrix, adjusts the migration mode according to the changes of the surrounding environment and itself, and changes flexibly in the three modes [8, 9]. Effective migration requires the coordinated dynamics of the cellular components involved, and these structures are strictly regulated by several signals [10]. To date, vital proteins related to cancer cell migration have been identified to include RhoA, RhoB, RhoC, Cdc42, Rac, and other members of the Rho GTPase family [10–12], the Ras superfamily [13, 14], the WASP/WAVE family [15–19], the Scrib complex [20–23], and Par complex [24, 25], PI3Ks [26] and PTEN [27, 28], PKCs [29], FAK [30, 31], ERK [32], and Src [33, 34], among others. Herein, the role of RhoC in cancer cell migration is of particular interest. RhoC reportedly affects cell movement by influencing the activities of actin and myosin, and cell adhesion [35], thereby affecting the process of cancer metastasis. According to the existing literature, it plays an important role in many cancers, such as influencing angiogenesis in bladder cancer [36] and tumorigenesis and epithelial-mesenchymal transition (EMT) in osteosarcoma [37], regulating the emergence of radioresistance in cervical cancer [38], and affecting prostate cancer treatments that target the glutamine pathway [39]. Certainly, its most significant role is promoting metastasis in breast [40–42], gastric [43, 44], colon [45, 46], bladder [47], prostate [48, 49], lung [50], pancreatic [51], liver [52] and other cancers. A recent study on prostate cancer has excitingly announced that vaccination against RhoC could potentially delay or prevent cancer recurrence and metastasis [53]. Additionally, murine studies have shown that RhoC is not essential to embryogenesis, but essential for metastasis, making it a possible target for gene modification [54]. To equip researchers with a more intuitive and detailed understanding of RhoC-related migration, this paper reviews the specific mechanism of RhoC involvement in each process or mode of migration. It also discusses the direction of future research on RhoC and its prospect as a target for anti-metastasis therapy.

## Main text

### The Rho GTPase family

**Fig. 1:**
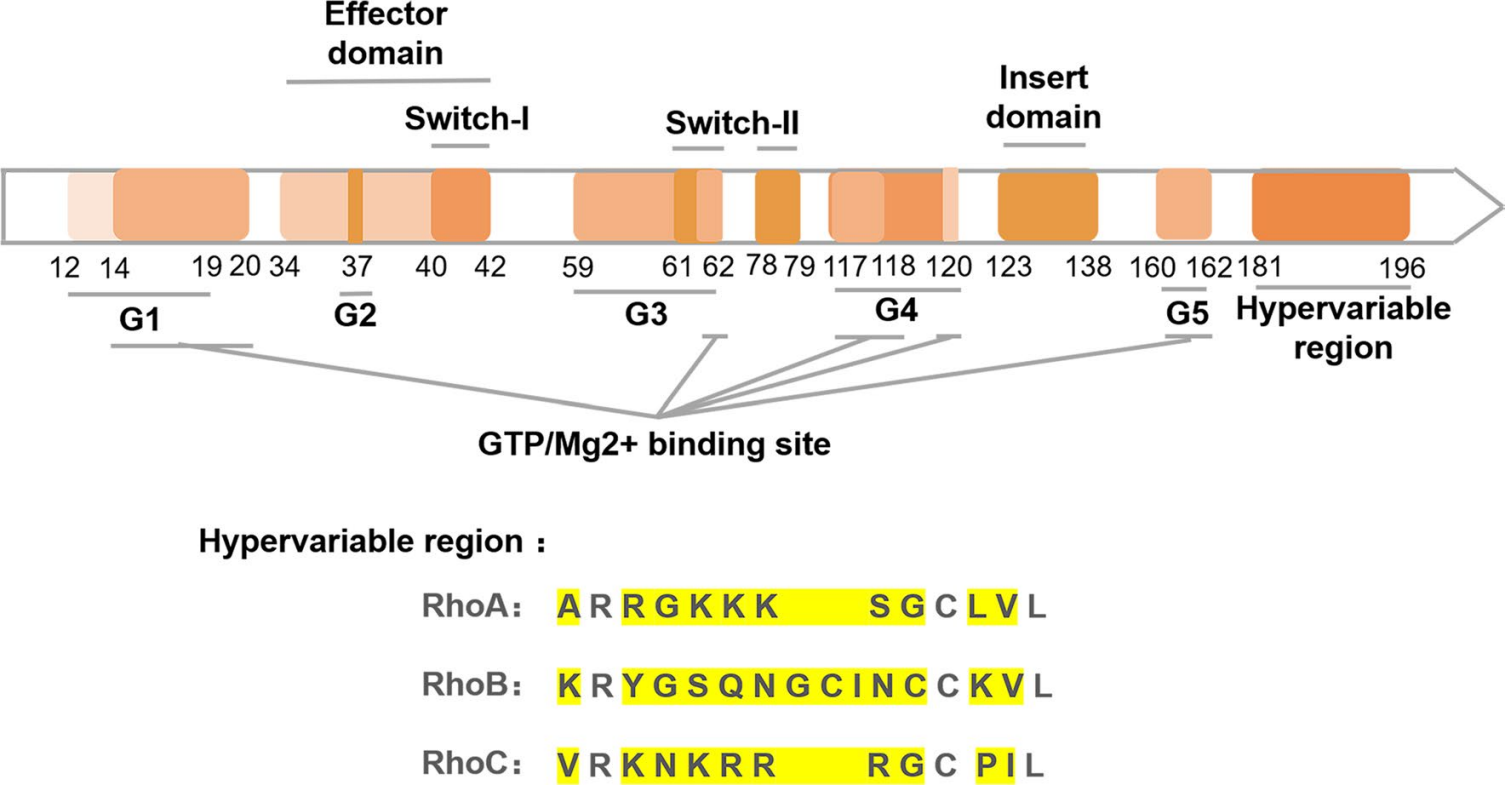
Rho GTPase domain organization. The sequence comparison of domain structures and hypervariable C-terminal regions of RhoA, RhoB and RhoC is shown. The yellow highlight represents the amino acid differences of RhoA, RhoB and RhoC in the hypervariable region

Rho GTPases are small signaling G proteins—also known as small GTPases, small G-proteins, or the Ras superfamily—that regulate the cytoskeleton, affect cell mobility, polarity and division, and play an important role in cell migration and invasion [55], as seen in Table 1. In humans, this family consists of twenty members, divided into eight different subfamilies and classified as either classic (typical) or atypical [56]. Classical Rho GTPases, including Rac, Rho, Cdc42, and the RhoF/RhoD subfamilies, are regulated by Rho-specific guanine nucleotide exchange factors (GEFs) and GTPase activating proteins (GAPs), which drive the switch between GTP- and GDP-bound states. GEF catalyzes the transition between GTP and GDP, thereby activating GTPase, whereas GAPs increase the intrinsic GTP hydrolysis rate of GTPases, thereby inactivating them [55, 57]. Atypical Rho family members include Rnd, RhoBTB, RhoU/V, and RhoH subfamilies, which are not regulated by GEF or GAP, but mainly modulated by GTP and other mechanisms, including post-translational modification [58]. In recent years, the role of the Rho subfamily of Rho GTPases in cell migration, has been widely studied. The Rho subfamily—which includes the highly homologous RhoA, RhoB, and RhoC that share 85% amino acid sequence identity—is known to regulate actin skeleton dynamics [59], as seen in Fig. 1. Like other GTPases, these Rho isoforms have intrinsic GTPase activity and shuttle between the inactive GDP-bound and active GTP-bound states [60]. However, many studies have shown differentiation of their intracellular distribution and function. For example, RhoA and RhoC are located in the cytoplasm, while RhoB is located in endosomes [61]. RhoA plays a key role in regulating the actomyosin contractility and cell proliferation, whereas RhoB regulates the transport of cytokines and cell survival, and RhoC is more important in cell movement [59]. Each step in the process of cell migration is guided and regulated by a variety of signal molecules. The Rho GTPase family is the most important of these regulators, among which RhoC has been proven responsible for cytoskeleton recombination and cell movement [35]. In this article, we focus on the specific mechanism by which RhoC influences the process of cell migration.

**Table 1:** The Rho GTPase family

Rho GTPase	Subfamily	Intracellular distribution	Major functions in cell migration	References
RhoA	Rho	plasma membrane; cytoplasm; nucleus;	Regulate contractility in the cell body; Regulate membrane protrusion;	[174–176]
RhoB	Rho	plasma membrane; endosomes; multivesicular bodies; nucleus;	Affect focal adhesion contacts with the substratum;	[177, 178]
RhoC	Rho	cytoplasm;	Restrict the breadth of lamellipodia;	[108, 179]
Rac1	Rac	plasma membrane; nucleus; mitochondria;	Stimulate the formation of lamellipodia;	[180, 181]
Rac2	Rac	plasma membrane;	Regulate actin remodeling; affect membrane ruffling;	[182, 183]
Rac3	Rac	perinuclear;	Stimulate the formation of lamellipodia; affect membrane ruffling;	[184, 185]
RhoG	Rac	plasma membrane; perinuclear;	Regulate the formation of membrane ruffles, lamellipodia, filopodia, and microvilli;	[186]
Cdc42	Cdc42	plasma membrane; the Golgi apparatus;	Regulate the formation of filopodia	[187, 188]
RhoJ	Cdc42	plasma membrane; organelle membrane;	Regulate the numbers, size and disassembly of focal adhesion;	[189, 190]
RhoQ	Cdc42	plasma membrane; endomembrane compartments;	Regulate membrane ruffling and stress fiber;	[191, 192]
RhoD	RhoD/F	plasma membrane; endosomes; the Golgi apparatus;	Regulate the formation of filopodia and membrane ruffling;	[193]
RhoF	RhoD/F	nucleoplasm; the Golgi apparatus;	Regulate the formation of filopodia;	[194–197]
RhoH	RhoH	plasma membrane; vesicles;	Regulate lamellipodium extension; Regulate migratory polarity;	[198, 199]
Rnd1	Rnd	plasma membrane; vesicles; cytoplasm;	Regulate cell adhesion and cell contraction;	[200, 201]
Rnd2	Rnd	cytoplasm;	Regulate cell contraction;	[202, 203]
Rnd3	Rnd	plasma membrane; cytoplasm;	Regulate tight junction integrity;	[202, 204]
RhoU	RhoU/V	plasma membrane; endomembrane compartments;	Regulate focal adhesion turnover, filopodia and cell adhesion;	[205, 206]
RhoV	RhoU/V	plasma membrane; endomembrane compartments;	Regulate filopodia and lamellipodia; regulate the formation of focal adhesion;	[146, 207, 208]
RhoBTB1	RhoBTB	Vesicular;	Do not regulate the actin cytoskeleton directly;	[146, 209, 210]
RhoBTB2	RhoBTB	Vesicular;	Do not regulate the actin cytoskeleton directly;	[146, 209, 210]

### History of RhoC

RhoC was first discovered in 1985, when Madaule and Axel isolated a new *Ras* gene family from the cDNA library of the *Aplysia* abdominal ganglion, naming it the *Rho* gene. It has significant homology with the *Ras* oncogene family and encodes a protein that shares 35% amino acid resemblance with H-ras [62]. The same research team proceeded to isolate two *Rho* family members from *Saccharomyces cerevisiae* and characterized them using DNA sequence analysis. These yeast genes, named *Rho1* and *Rho2,* respectively shared 70 and 57% identity with those of the marine snail *Aplysia*, and 53% identity with each other. Furthermore, knockdown of these genes revealed that, contrary to *Rho2*, *Rho1* was required for cell activity [63]. The authors further examined the *Rho* gene in humans and rats, and suggested that the human *Rho* gene potentially consisted of three members [64]. However, due to lack of biological findings, little attention was paid to these discoveries. Then, Yeramian et al. published the nucleotide sequence of human *Rho* cDNA clone 12 [65], followed by publishing of the coding sequences of clones 6 and 9 the year after, by Chardin et al. Clones 12, 6, and 9 were named RhoA, RhoB, and RhoC, respectively [66]. Chardin et al. further found that the bacterially expressed product of the human *RHOC* gene is ADP-ribosylated by *Clostridium botulinum* C3, and corresponds to the C3 substrate of eukaryotic cells in size, charge, and behavior. When Vero cells were treated with C3, their microfilaments disintegrated and radial symmetry morphology changed, but no direct effect on actin was evident. Therefore, they speculated that the unmodified form of Rho protein may be involved in regulation of the cytoskeleton [67]. This hypothesis was confirmed by Stasia et al. who found that microinjection of exonuclease C3 caused significant changes in the actin filament network of 3T3 cells, including significant inhibition of neutrophil movement and disorder of actin filament assembly, which was explained as the result of ADP-ribosylation of Rho protein [68]. Morris et al. subsequently used fluorescence in situ hybridization to map the *RHOC* gene to the p13-p21 band on chromosome 1 [69]. Since then, mounting RhoC-research has focused on its effect on the actin skeleton.

### Transcriptional regulation and mutation of RhoC

Few studies have focused on regulating RhoC gene expression at the transcriptional level. In one such study, activated p53— when subjected to genotoxic stress—directly binds to the regulatory element located in the intron 2 region of the RhoC gene, inducing RhoC expression [70]. In addition, ETS Proto-oncogene 1 (Ets-1) transcription factor binds to the promoter and stimulates expression of RhoC during EMT, in colon cancer [71]. In hepatocellular carcinoma (HCC) infected with hepatitis B virus (HBV), HBs and HBx proteins induce the expression of Ets-1 transcription factor, thereby enhancing the activity of the RhoC promoter to upregulate RhoC expression [72, 73]. Zhou et al. found that the binding of endogenous HIF-3α to the RhoC promoter under hypoxia, increases the RhoC mRNA level and promotes cancer cell invasion [51].

MicroRNA (miRNA) regulates gene expression at the post-transcriptional level. Two studies have demonstrated that miR-138 inhibits the migration and invasion of tongue squamous cell carcinoma (TSCC) and head and neck squamous cell carcinoma (HNSCC) cells, respectively, by targeting RhoC mRNA [74, 75]. Liu et al. showed that miR-372 overexpression reduces the expression of RhoC through its three prime untranslated region (3′UTR) and inhibits the proliferation, migration, and invasion of endometrial adenocarcinoma (EC) cells [76]. Many studies have shown that miR-10b inhibits homeobox D10 (HOXD10) in colorectal cancer, metastatic breast cancer, and malignant glioma cells, respectively, resulting in increased expression of RhoC [77–80]. In ovarian cancer, miR-519d directly binds to and inhibits the expression of the 3′UTR of RhoC mRNA. The negative correlation between miR-519d and RhoC is also demonstrated in a xenotransplantation model in nude mice^81^. Zhou et al. showed that miR-493 directly targets RhoC, resulting in a significant decrease in its mRNA and protein expression, and inhibits the growth, invasion, and metastasis of gastric cancer cells [82]. In HCC and epithelial ovarian cancer, miR-106b enhances cell migration by inducing RhoC expression [83, 84]. In a recent study, miR-302e targets circRhoC—a putative circular RNA (circRNA) emerging from RhoC mRNA—as a tumor suppressor [85]. Xie et al. proved that miR-455 specifically recognizes the 3′UTR of RhoC and inhibits both RhoC expression and the proliferation of hepatoma cells, and promotes cell apoptosis [86]. The latest research shows that the downregulation of miR-17-5p in esophageal cancer cells, leads to the upregulation of RhoC, which is the direct downstream target of miR-17-5p [87]. The role of miRNA in regulating RhoC expression is indicative of the potential use of this mechanism in developing new cancer treatment schemes. For example, Shao et al. proposed a tumor-triggered personalized miRNA cocktail therapy to treat HCC, by encapsulating miR-199a/b-3p mimics (miR199) and antimiR-10b (antimiR10b) into PCACP—the polymer-based nanoplatform, PEI-βCD@Ad-CDM-PEG—and significantly inhibiting cell proliferation and tumor growth [88].

Overexpression of the RhoC gene is associated with the progression of pancreatic^89^, liver [90], breast [91], and many other cancers. However, according to current research, RhoC itself does not seem to mutate, but rather promotes cancer metastasis through its overexpression [92]. In a past mutation study, RhoC-43 V (mutated to valine at position 43) is more effective in driving the invasion of ovarian cancer cells, than wild-type RhoC [93]. Another study showed that the dominant negative mutant of RhoC significantly reduces the actin polymerization induced by myosin-interacting guanine nucleotide exchange factor (MyoGEF), and inhibits the polarity and invasive activity of breast cancer cells [94]. In addition, when the arginine residue at position 188 of the polybasic region (PBR) of RhoC is replaced with other amino acids, RhoC membrane localization is significantly inhibited. The consequent RhoC activity is poor, resulting in decreased motility of cancer cells [95].

### 2D migration

**Fig. 2:**
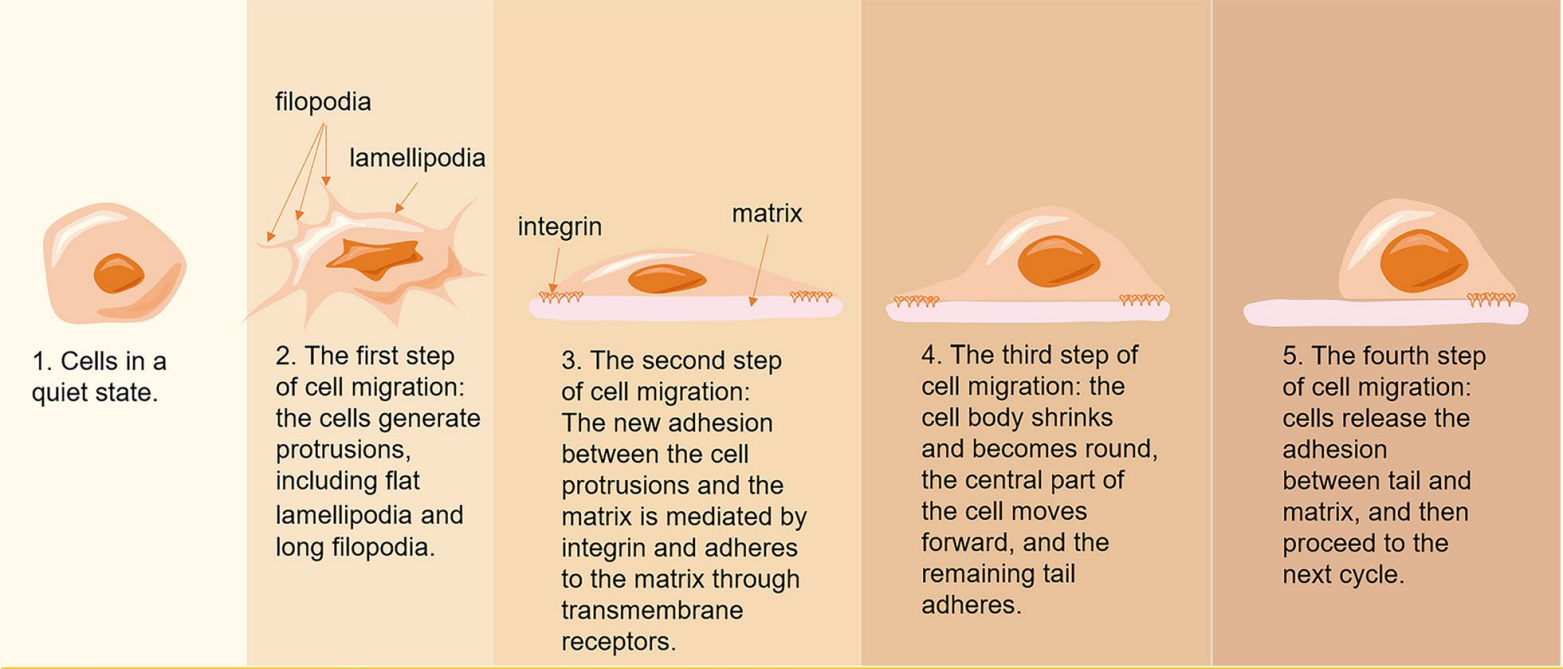
Four-step cycle of cell migration

The classical cell migration model (Fig. 2) describes the process of cell migration in terms of four perpetual steps that include 1. Morphological polarization: this includes the formation of lamelli- and filopodia [96]. 2. Formation of new adhesions: in the process of single cell migration, this mainly entails the formation of new cell–matrix adhesions, which is usually driven by actin polymerization and adherence to the ECM through transmembrane receptors [97, 98]. In the process of collective cell migration, intercellular adhesion helps to coordinate the activities of adjacent cells, resulting in more persistent migratory behavior [99]. 3. Contraction of the cell body: a myosin motor in each moving cell pulls on actin filaments that retract the trailing end of the cell due to the generated contractile forces, moving the cell body forward [100]. 4. Release of trailing adhesions: the adhesion between the trailing end of the cell and the surrounding matrix is dissociated, whereby the cell moves forward [101].

#### Morphological polarization

**Fig. 3:**
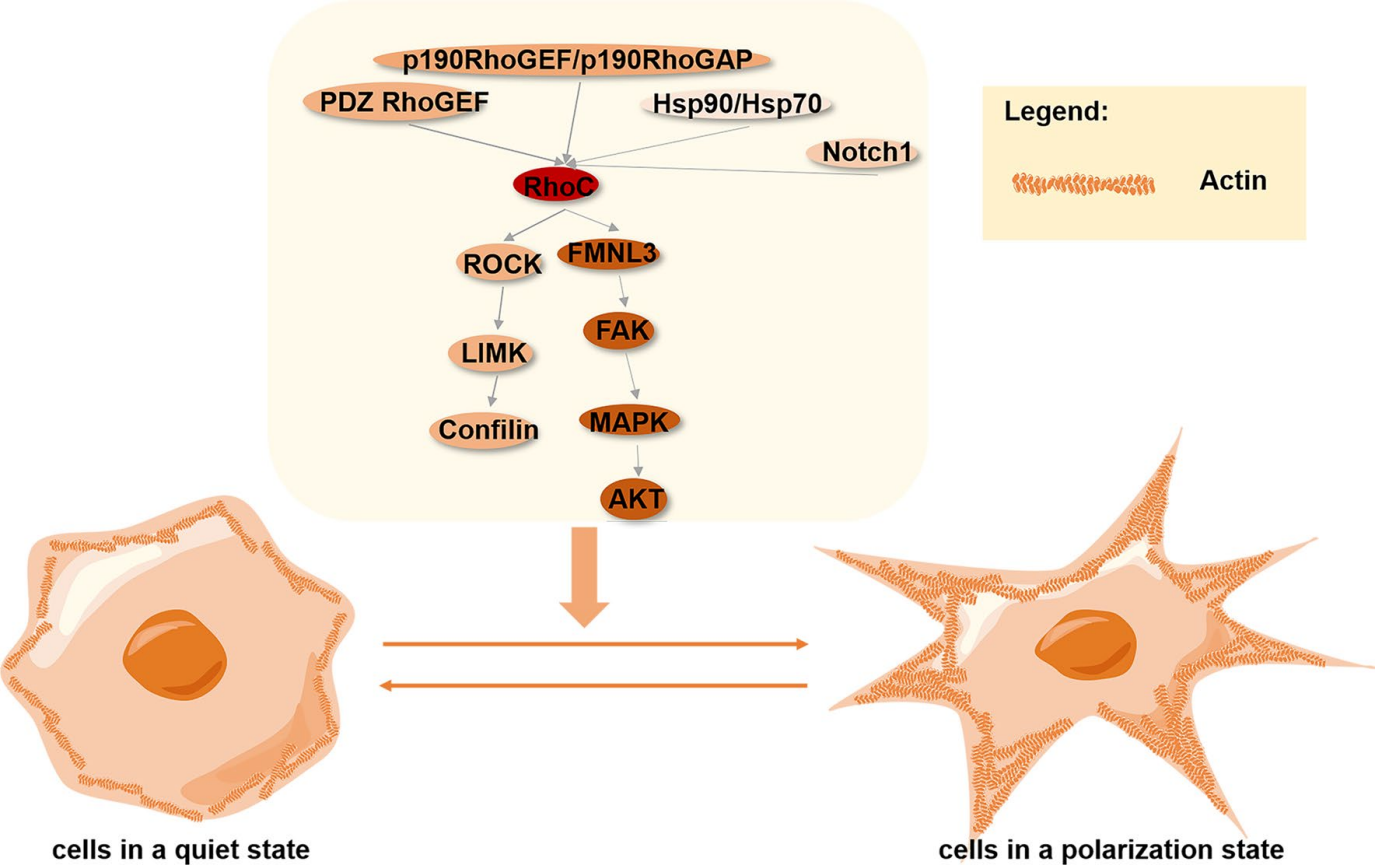
The role of RhoC in polarization. RhoC is regulated by upstream PDZ-RhoGEF [112, 113], p190RhoGEF/p190RhoGAP [111], Hsp90/Hsp70 [114] and Notch1 [110]. After activation, the downstream ROCK/LIMK/cofilin [114–118] and FMNL3-regulated FAK/MAPK/AKT signaling pathways [104] are activated, resulting in a change in cell morphology to a multi-protuberant polarized structure. When the RhoC signal is blocked, the cell presents with a dispersed morphology [108, 109]

The first step in cell migration is the formation of projections—mainly lamelli- and filopodia—from the leading edge of the cell, the location of which determines the direction of migration [96, 102]. RhoC can regulate the formation of protrusions in many cells, and its expression and regulation has been proven in human macrophages, as well as prostate cancer, colorectal carcinoma, and breast cancer cells, respectively [103, 104]. During cell migration, myosin II activity inhibits the formation of frontal protrusions [105]. However, RhoC plays a role in limiting myosin II activity, allowing cells to form the projections required for migration [105, 106]. RhoC expression and activation are further required for directional migration and invasion, as it seems to be effective in limiting lamellipodial broadening [60, 107]. When RhoC is depleted, cells will form abnormally wide lamellipodia with multiple branches due to defective actin polymerization in the leading edge of cells. These branches cannot effectively penetrate the ECM, resulting in reduced migration rate and persistence [108, 109]. Gou et al. proved that ectopic RhoC overexpression can enhance the formation of lamellipodia in ovarian carcinoma cells [110]. Another study used MTLn3 cells—a highly metastatic rat mammary adenocarcinoma cell line—to demonstrate that p190RhoGEF and p190RhoGAP act as upstream regulators that limit RhoC activation in specific regions, influencing the formation of specific functional protrusions, in the regulation of migration-related processes [111]. Coincidentally, PSD-95/Discs-large/ZO-1 homology (PDZ)-RhoGEF is also involved in RhoC activation in ovarian carcinoma [112, 113]. Moreover, Willmer et al. proved that heat shock proteins 90 and 70 (Hsp90/Hsp70) organizing protein (Hop) is another upstream molecule affecting RhoC, and it is co-localized with actin in lamellipodia. Knockdown of Hop results in decreased RhoC levels in breast cancer cells, notably inhibiting the formation of filopodia [114]. Traditionally, RhoC is thought to regulate the migration phenotype through the Rho-associated protein kinases (ROCK) 1 and 2. However, Vega et al. found that neither ROCK1- nor ROCK2-depletion phenotypes resemble the RhoC-inhibition phenotype, inferring that RhoC, at least partially, does not regulate cell morphology through ROCK1 or ROCK2. Furthermore, although ROCK2 and RhoC-knockdown both reduce directional migration, they seem to work by different mechanisms: RhoC-knockout cells have broad lamellipodia, while ROCK2-knockout cells have narrow protuberances [108]. Moreover, Formin-like protein 3 (FMNL3) reportedly interacts directly with RhoC in vivo and in vitro, is co-localized in the cytoplasm, activates the downstream signal transduction of FAK/MAPK/Akt, and limits the widening of the lamellar membrane to promote cell polarization and migration [104]. In addition, Vega et al. identified FMNL3 as a new RhoC-specific target, and showed that RhoC promotes polarized migration in PC3 prostate cancer and MDA-MB-231 breast cancer cells, respectively, through FMNL3. Expression of FMNL3 rescues the broad lamellipodial phenotype induced by RhoC knockdown [108]. Inactivation of cofilin—a protein that promotes actin polymerization—is an important step in its activity cycle. RhoC activation can trigger the ROCK/Lin11/Isl1/Mec3 kinase (LIMK) pathway to phosphorylate and inactivate cofilin, thereby regulating the actin cytoskeleton to regulate human glioma, squamous cell carcinoma, and breast cancer cell migration [114–118]. EMT is also closely related to the migration of cancer cells. Sequeira et al. illustrated that RhoC inactivation induces morphological changes commensurate with EMT, accompanied by increased random motility and decreased directional migration, in PC3 prostate cancer cells [119]. In cervical cancer cells, Notch homolog 1, translocation-associated (*Drosophila*) (Notch1) and RhoC make similar phenotypic contributions to EMT, and Notch1 inhibition can reduce RhoC activity, suggesting that the latter plays a role as an effector of the former [110]. These processes are summarized in Fig. 3.

The role of RhoC in limiting lamellipodia broadening is clear. Although more than one specific regulatory pathway has been found, they have not been clearly studied. Also, the mechanism may not be limited to the above regulatory methods, and the molecules or pathways interacting with RhoC have not been fully scrutinized. For example, IQ motif-containing GTPase-activating protein 1 (IQGAP1) is also associated with cell migration [120, 121], and has been proven to be an effector of RhoC in promoting the migration of breast cancer [122] and gastric cancer cells[44]respectively; it is also observed at the leading edge of migrating cells [122]. Coincidentally, RhoC is also enriched in the leading edge of migrating cells [123]. We have reason to believe that RhoC can regulate protrusion formation through IQGAP1, but strong experimental proof is lacking, as many similar molecules’ functions have not been specified. Future research should pay more attention to this matter.

#### Regulation of cell adhesion

Effective cell migration requires precise regulation of cell adhesion, including cell–matrix adhesion and cell–cell adhesion.

In the process of single cell migration, cells need to continually form new adhesions between the cellular projections and the matrix, to anchor the cells; this is called cell–matrix adhesion [124]. To enable cell movement, the formation and dissociation of cell–matrix adhesions needs to be repeatedly cycled [125]. At the leading edge of a cell, lamellipodia form an adhesive force that connects the ECM to the actin skeleton, thereby anchoring the protrusion [126]. Actin stress fibers in the projections connect the cells to the matrix, thus providing traction for leading edge advancement and whole cell body displacement [106]. In a study of inflammatory and aggressive breast cancers, the role of RhoC in regulating cell–matrix adhesion is particularly important, as the response to matrix adhesion signals transmitted by integrin—by promoting the assembly of adhesion spots and stress fibers—is a key function of the Rho protein [127]. Bravo-Cordero et al. observed in MTLn3 cells, that RhoC activity is enriched in the area behind the front edge of a cell, and proved that RhoC activation increases the cofilin level in a ROCK-dependent manner, thereby promoting the formation of cellular protrusions in the migratory direction. The spatial localization of RhoC activity can be used as a directional compass to limit the polymerization position of actin by limiting the activity of cofilin, to determine where the protrusion should form [123]. To determine which molecules are related to signal conduction downstream of RhoC during the regulation of matrix adhesion, microarray analysis was conducted on MCF10A breast epithelial cells in which RhoC had been overexpressed. It was found that RhoC-overexpression significantly increased the mRNA level of fibronectin and Caveolin-2, as well as the migration ability of the cells [128]. Moreover, Caveolin-1 interacts with RhoC in pancreatic cancer, bladder cancer, and inflammatory breast cancer cells, respectively^129^–^131^. Furthermore, in migrating pancreatic carcinoma cells, the RhoC C-terminus domain co-localizes with, as well as enhances the activation and circulation of integrin α5β1, in addition to reducing cell adhesion and promoting cell migration. Src in melanoma cells is also involved in processes that occur downstream of RhoC engagement with integrin α5β1 [132, 133]. Low expression of NADPH oxidase 4 (*NOX4*) is related to decreased cell–matrix and cell–cell adhesions. Crosas-Molist et al. described for the first time that low expression of NOX4 leads to high expression of RhoC in HCC, which weakens the adhesion between cells and ECM, resulting in increased cell migration and invasion [134]. The Rho-specific GEF, Tumor endothelial marker 4 (TEM4), is an important regulator of the actin cytoskeleton that modulates cell–matrix adhesion by signaling to RhoC. The depletion of TEM4 and RhoC leads to increased cell–matrix adhesion and decreased cell migration in endothelial cell, which is also affected by ROCK [106].During migration, matrix adhesion is not only weakened, but also a dynamic cycle of formation and dissociation. By constantly updating the adhesion sites, the cells move forward. The regulation of RhoC on integrin is in line with this process. The current literature only shows the inhibitory effect of *NOX4* on RhoC, but whether there is another link between *NOX4* and RhoC remains to be determined.

**Fig. 4:**
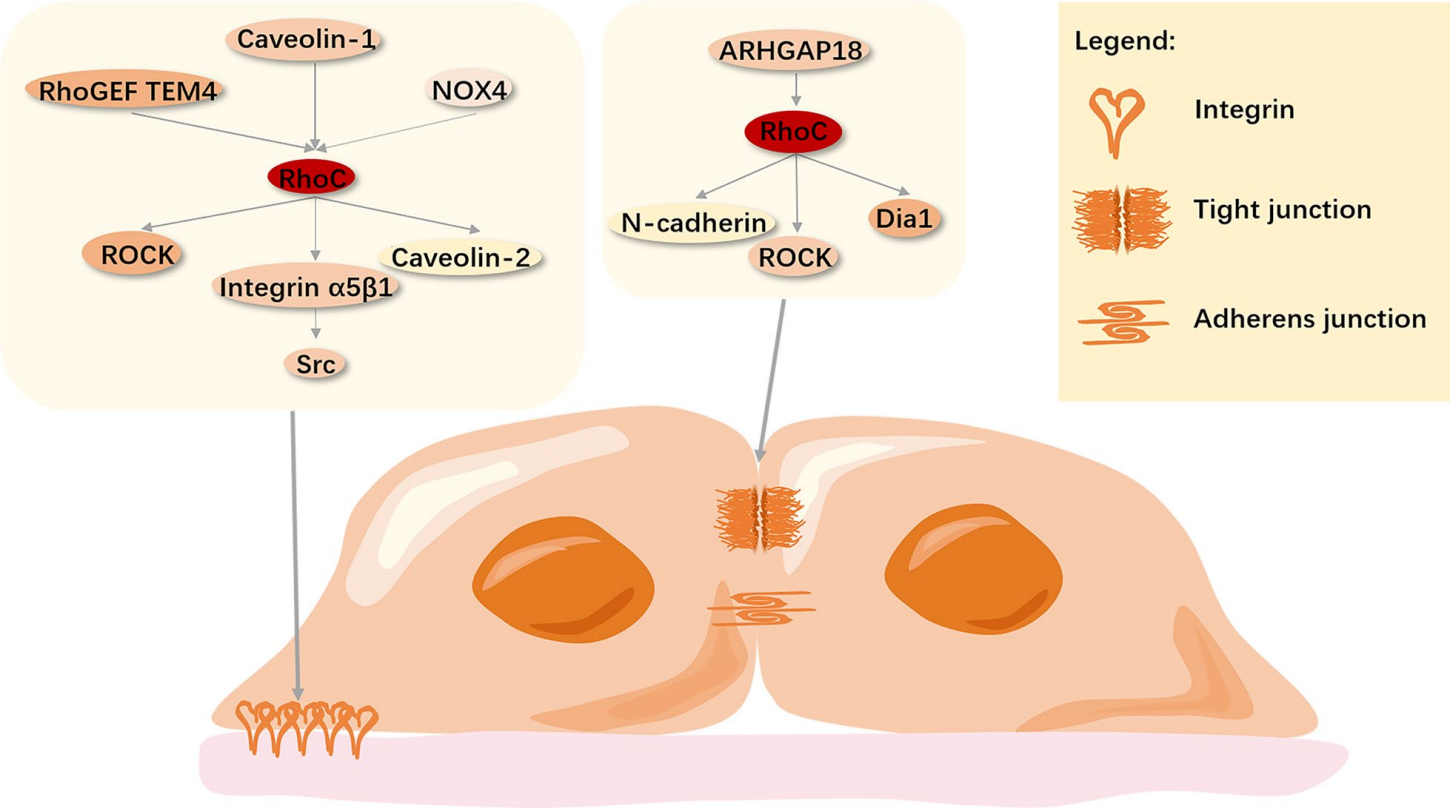
Role of RhoC in adhesion regulation. Cell–matrix adhesion: RhoGEF TEM4 [106], Caveolin-1 [129–131], and *NOX4 *[134] regulate the activity of RhoC that, in turn, activates ROCK [106], integrin α5β1 [132, 133], and Caveolin-2 [128]. These factors respectively weaken the adhesion between cells and matrix, increase the adhesion cycle, and promote cell migration behavior^127^

Cell–cell adhesion is mediated by four main types of junctions, namely adherens, tight and gap junctions, and desmosomes [7]. Dependent on the different cell types and tissue environment, cells retain intercellular adhesion when they migrate collectively, which enables the cells to interact with each other and alter cell polarity. The process of single-cell migration requires the loss or weakening of intercellular adhesion [135]. Studies have shown that cells inhibited by RhoC showed a tight junction tissue structure, indicating that RhoC regulates both cell–matrix and cell–cell adhesion, in the migration cascade [127]. RhoC mainly regulates two junctional complexes, namely tight (TJs) and adherens junctions (AJs)—constituting the endothelial barrier—between cells. In one study, it was found that RhoC is localized and activated at the endothelial junction in primary human endothelial cells, resulting in the destruction of intercellular junctions [136]. AJs connect the actin cytoskeletons of adjacent cells and depend on the homogenous binding of cadherins, including E- and N-cadherin. The expression and localization of E-cadherin are deranged by activated RhoC in prostate cancer cell [137]. Similarly, RhoC and ROCK mediate the breakdown of TJs and AJs in endothelial cells [59]. Rho GTPase activating protein 18 (ARHGAP18) is a type of RhoGAP that specifically acts on RhoC; its knockdown leads to RhoC-overexpression, which directly leads to the destruction of ROCK-dependent AJs in fibroblasts and human umbilical vein endothelial cells (HUVEC) [59, 138, 139]. Studies have shown that RhoC and Mammalian Diaphanous 1 (mDia1) are co-localized in migrating cells, and depletion of the latter results in a phenotype similar to what is seen in RhoC depletion. RhoC-overexpressing cells can rapidly break and recombine AJs, resulting in highly dynamic cell behavior. Downstream of RhoC, both mDia1 and ROCK are involved in the regulation of AJs [140]. RhoC maintains AJ stability through mDia1 and antagonizes the destabilization of ROCK-mediated intercellular adhesion destruction, which is consistent with the synergistic effect of the two pathways necessary for RhoC signaling [140–142]. Figure 4 provides a graphical summary of these data.

The dynamic regulation of cell adhesion ability is essential to the stability of migration cycle. In this process, RhoC mediates the destruction of tight connection and adhesion connection through different mechanisms. The most important is the role of ROCK and mDia1, which are controlled by each other and play the opposite role.

Cell–cell adhesion: tight junctions and adherens junctions are involved in cell migration [59]. ARHGAP18^59^,^138^,^139^ regulates the activities of RhoC and downstream ROCK [59], N-cadherin [137], and mDia1 [140], resulting in the dynamic behavior of cells.

#### Contraction of the cell body

**Fig. 5:**
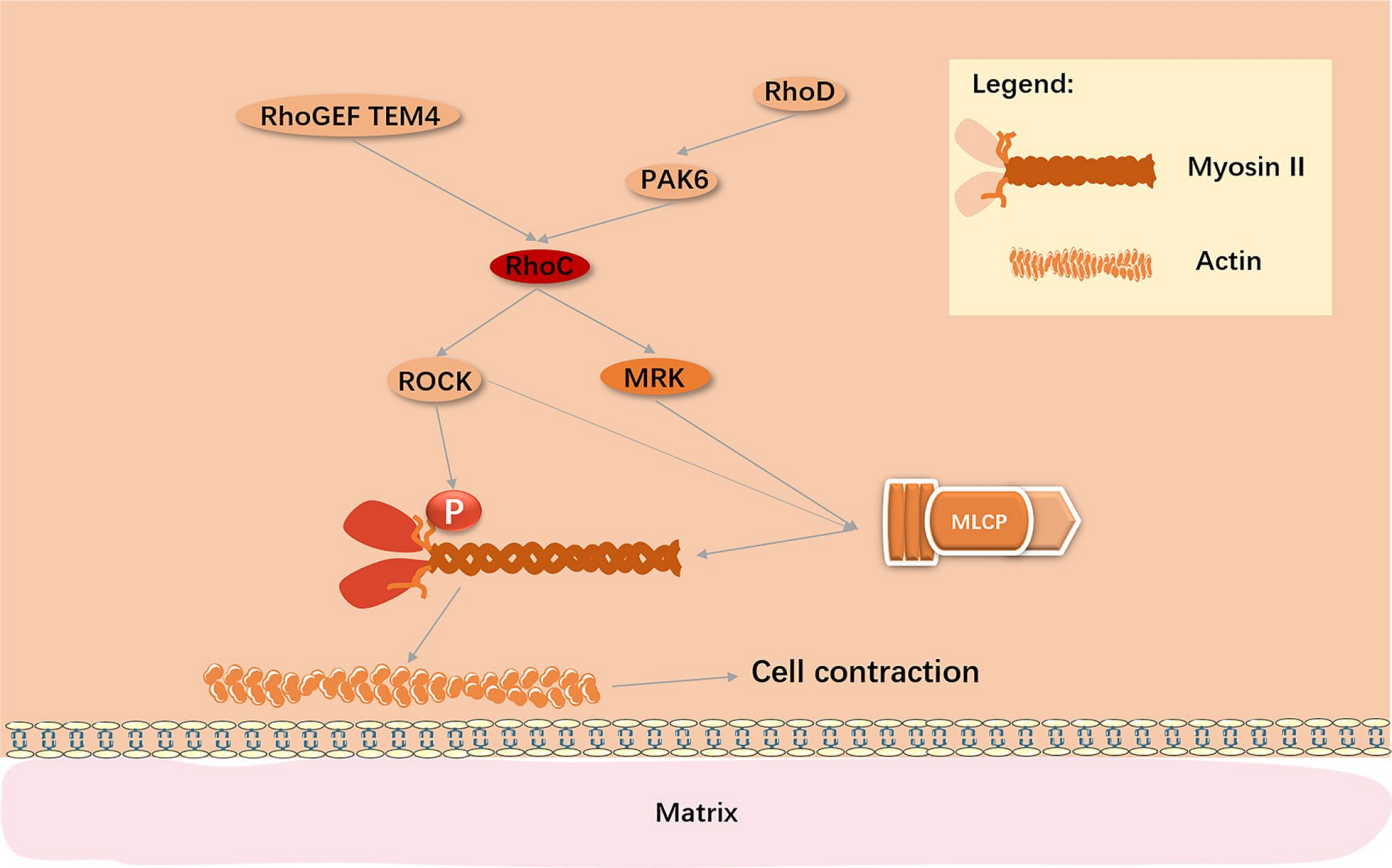
RhoC regulates cell contraction. RhoC is regulated by RhoGEF TEM4tem4 [106] and RhoD/PAK6 [147] in the process of cell contraction, in which RhoC plays a dual role, by first regulating the redistribution of myosin II through ROCK [148], and then managing the activity of MLCP through ROCK and MRK [149, 150], to modulate myosin and regulate cell contraction

In the process of migration, cells can produce an active pulling force through contraction of actin and myosin, to make cells move forward [143]. The contractile apparatus in cells consists of F-actin and myosin II. Phosphorylated myosin light chain (MLC) promotes the contraction of myosin, facilitating its interaction with F-actin to produce a contractile force. In a migrating cell, contractile force is usually applied to focal adhesions to destroy the adherence and cause cell contraction [100]. The key factor of assembly and contraction of actin and myosin is the activation of myosin II by MLC phosphorylation, mediated by ROCK [144]. Moreover, ROCK indirectly increases myosin II activity by inhibiting MLC phosphatase (MLCP) [145]. Although ROCK can be simultaneously activated by RhoA and RhoC, research by Jackson et al. has shown that RhoA is not required for the formation of actin stress fibers, the assembly of which can be induced by both active RhoA and RhoC [145, 146]. Durkin et al. found that the ROCK signal mediated by RhoC can be expressed independent of the RhoA signal in both HeLa and human osteosarcoma (U-2 OS) cells, to drive the redistribution of myosin II. This promotes the contraction of cells, which can be suppressed by RhoD/PAK6 signaling [147]. However, the contraction due to increased ROCK-signaling cannot fully account for the activation of RhoC, which indicates that other effector proteins need to cooperate with ROCK [148]. In HeLa, U-2 OS, and human ovarian clear cell carcinoma (ES-2) cells, MLK-related kinase (MRK) also acts as an effector of RhoC and dynamically regulates myosin by controlling MLCP activity during cell migration [149, 150]. TEM4, which was discussed previously, not only regulates cell–matrix adhesion through RhoC, but also limits myosin II activity to control cell contraction [106]. The interactions responsible for cell contraction, are displayed in Fig. 5.

#### Tail retraction

To conclude the forward movement of the cell, it is necessary to extend the protrusion and retract the tail [138]. The migration cycle culminates in the release of posterior adhesions followed by tail retraction—that requires Rho kinase and is a myosin-dependent process^97^—resulting in a forward displacement of the cell body [151]. In some cells, the rate of backward release determines the overall migration rate. RhoA-mediated ROCK activation has been suggested to play an important role in this process[152] and it has been shown that ROCK1-depleted cells present with defective tail retraction, which is consistent with the phenotype of RhoA-knockout cells [151, 153]. RhoB-deletion appears not to affect tail length [154]. As mentioned, RhoC-deficient cells do not have long tails and show a diffuse phenotype. Therefore, current research outcomes seemingly indicate that RhoC does not control the post-release process of the migration cycle.

### 3D migration

**Fig. 6:**
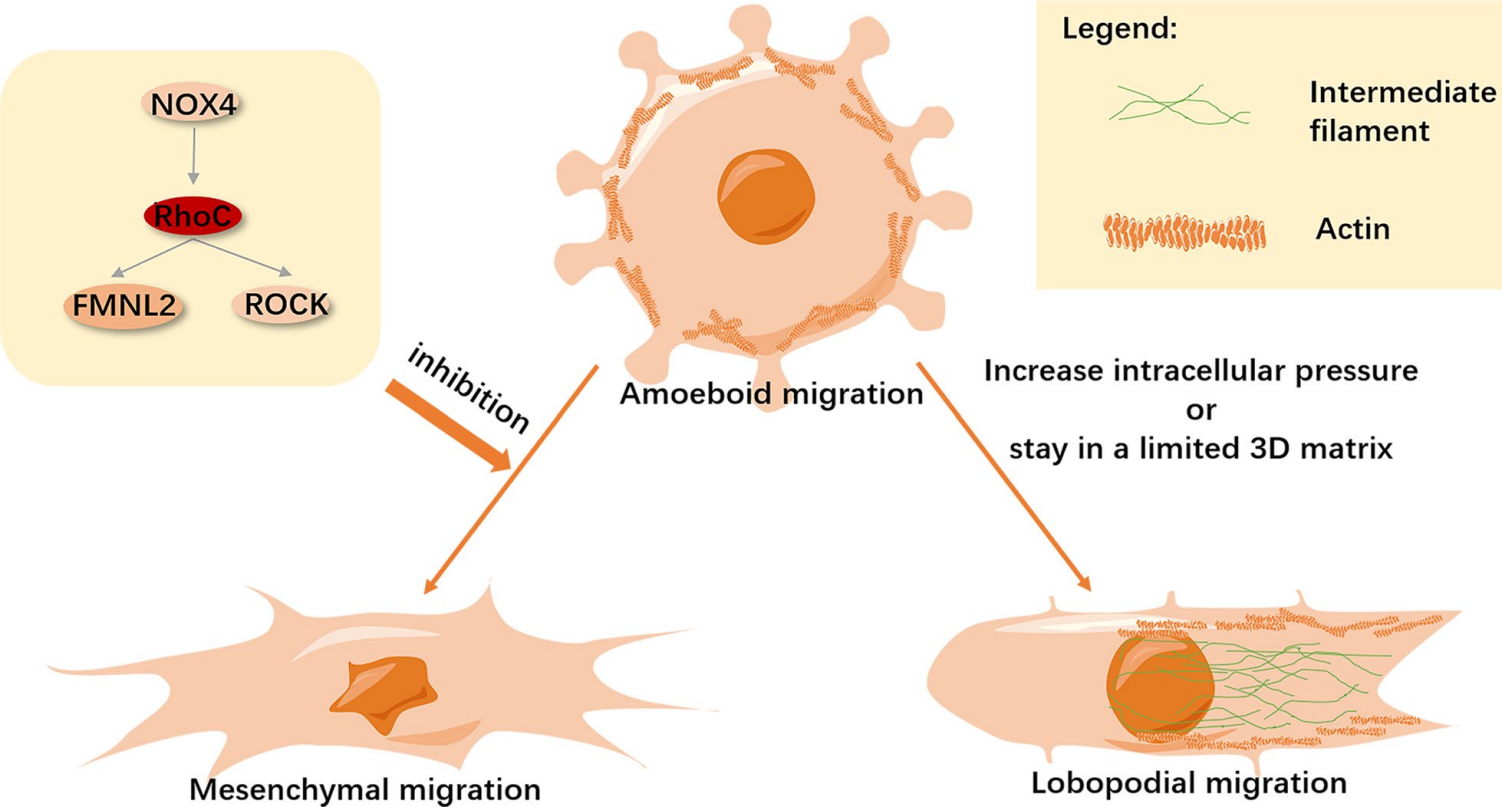
Switching of 3D migration mode. During 3D migration, changes in the surrounding environment can stimulate cells to switch between three migration modes^155^,^160^,^161^, which include amoeboid migration with blebbing as the main method [156, 162], in addition to mesenchymal, and lobopodial migration. Amoeboid migration is dependent on RhoC-signaling and cells switch to mesenchymal migration when the RhoC signal is blocked [134, 158]. However, when the cells reside in a limited 3D matrix, or the intracellular pressure increases, cells switch to lobopodial migration mode [2].

In 2D cell culture, cells have larger lamelli- and filopodia, whereas 3D cells have a less exaggerated appearance [155]. Cell migration in a 3D environment is closely related to the process of cancer metastasis, as cancer cells can use the 3D migration mode to invade their environment [156]. In a 3D environment, most cells move by three modes, namely amoeboid, lobopodial and mesenchymal (called lamellipodial) migration [157]. Three important factors regulating 3D cell migration patterns are cell–matrix adhesion, the Rho family of small GTPases, and protease [7]. Studies in 3D environments show that cancer cells switch between these invasion patterns according to the activation of specific Rho GTPases, during the process of metastasis [158, 159], as seen in Fig. 6.

#### Amoeboid migration

Amoeboid cell migration is similar to the movement of protozoal amoebae, which is characterized by round cells undergoing extensive deformation, and low-level adhesion. The migration strategies include contraction-based blebbing or entirely polymerization-driven gliding [163]. This form of vesicular migration—morphologically featured as movement facilitated by the formation of vesicles—is particularly useful for cell motility in a confined space. Activated myosin II increases local hydrostatic pressure, resulting in localized rupture of the actin cytoskeleton from the membrane. Resultantly, the membrane is rapidly pushed outward by cytoplasmic fluid flow, which promotes the formation of vesicles. Thereafter, actin polymerizes on the membrane to form a new actin cortex, promoting vesicle contraction [156]. High-resolution imaging demonstrated that RhoC-overexpressing cancer cells invade tissues by extending small membrane protrusions and vesicles—a typical amoeboid migration pattern—in a zebrafish xenotransplantation model^164^. High expression of RhoC in HCC cells significantly increases myosin II activity and the percentage of vesicles, whereas high myosin II activity and low adhesion are key to rapid amoeboid migration [165]. Lehman et al. demonstrated that RhoC-depletion reduces cell invasion in a lymphatic system invasion model used to study amoeboid movement in cancer metastasis [166]. ROCK—a classic effector of Rho GTPases—also plays a role in the regulation of amoeboid migration, and its affinity for RhoC is greater than for RhoA [115]. Kitzing et al. found that FMNL2 selectively interacts with RhoC and as an effector thereof, to regulate RhoC-dependent amoeboid migration. The expression of RhoC can partially alleviate the self-inhibition of FMNL2, indicating that activated RhoC regulates FMNL2 regulated actin dynamics [158]. The loss of *NOX4* can up-regulate RhoC expression and actin contractility, thereby promoting amoeboid migration [164].

#### Lobopodial migration

Lobopodial migration can be regarded as a combination of amoeboid and mesenchymal migration. When cells migrate in limited 3D matrix, they switch to lobopodial migration [167], where they are elongated and the cell membrane clings to the nucleus, dividing the cell into two parts [168]. Myosin II arcs, which are connected to vimentin intermediate filaments, produce tension that causes the nucleus to move forward in a piston-like manner, resulting in a pressure difference between the front and back ends of the cell. Resultantly, the front end produces lobopodial protrusions, forming new cell–matrix adhesions that circulate and cause the cell to move forward [169]. Research has shown that Rho, ROCK and myosin II constitute part of the lobopodial migration mechanism. The inhibitory effects of Rho and ROCK can switch lobopodial migration to lamellipodia-based 3D migration [170]. It has been observed in acute lymphoblastic leukemia cells that increasing intracellular pressure can also induce a change from low-pressure lamellipodial to high-pressure lobopodial migration [161], and increased intracellular pressure can be achieved by increasing the contractility of myosin in human foreskin fibroblasts (HFFs) [171]. Although current research does not indicate whether RhoC plays a role in regulating lobopodial migration, there is clear evidence that the RhoC-mediated ROCK signal can be induced independent of the myosin II-regulated RhoA signal [105, 106]. Therefore, it is reasonable to infer that RhoC also plays an indispensable role in regulating lobopodial migration, but there is a lack of relevant research to prove this.

#### Mesenchymal migration

The mesenchymal migration pattern is largely similar to that of 2D migration, in which the leading edge of a cell undergoes actin polymerization to produce lamelli- and filopodia [2]. The mesenchymal migration pattern is characterized by slender, spindle-shaped cells, and extracellular proteolysis. Notably, it has been observed in HT-1080 fibrosarcoma and MDA-MB-231 breast carcinoma cells, respectively, that the inhibition of pericellular proteolysis gives rise to a migration mode switch from mesenchymal to amoeboid movement [172]. Additionally, Rho GTPases must be inhibited during mesenchymal migration, as active Rho will cause cells to change into a Rho-dependent amoeba-like migration mode [173]. Although the literature does not explain the role of RhoC in mesenchymal migration, we believe that it is indispensable in the regulation of this process.

## Conclusion

Migration plays an important role in the occurrence and development of many physiological and pathological processes. The regulation of migration is a complex process involving many molecules and a wide range of mechanisms. In depth understanding of migration can help us tackle cancer metastasis, embryonic development, and immune response. In this paper, we reviewed the role of RhoC—a classic member of the Rho family—in both typical 2D, and newly proposed 3D migration models. Since its discovery, it has been difficult to develop unique, RhoC-specific small molecule inhibitors or activators, due to the high level of homology between RhoA, RhoB, and RhoC as well as the emergence of RhoC as the whole Rho subfamily. Later technologies, such as siRNA, enabled researchers to discover the unique effects of RhoC that were independent of RhoA and RhoB. In recent years, with increased understanding of cell migration mechanisms, the major impact of RhoC on cell motility has become evident. In the classic 2D cell migration cycle, all processes, except for tail retraction, are precisely regulated by RhoC. In the emerging 3D migration theory, amoeboid migration is also known as the Rho-dependent migration model, in which the important RhoA-independent role of RhoC has been proven. Studying RhoC functionality can contribute to a better understanding of cancer metastasis, as well as aid in development of new anti-metastasis therapeutic targets. Cancer metastasis has been a major challenge for the past century, the solution to which requires deeper and wider understanding of its internal mechanism. The significant role of RhoC in tumor metastasis suggests that it may have great potential as a target for anti-metastasis therapy. At present, anti-cancer research involving RhoC is not very extensive, possibly due to insufficient availability of detail concerning the mechanism of RhoC action in cancer metastasis. However, existing studies clearly indicate that both drugs and vaccines against RhoC have achieved excellent results. Perhaps RhoC-targeted treatment schemes will be accepted into clinical practice in future. Further to the pathological process, RhoC-controlled amoeboid migration is also of great significance in many physiological processes. The functions of RhoC are very diverse, but due to the limitations of current technology and the development of specific inhibitors, our understanding of RhoC is still limited, and it is difficult to accurately locate RhoC in related studies, which limits the independent exploration of RhoC. In addition, research on 3D migration is more complex than 2D migration, with a lack of convenient and controllable detection methods, thereby hindering research progress. The mechanisms of RhoC, such as how RhoC plays a role in lobopodial migration and mesenchymal migration, remain unclear and warrant further investigation.

## Data Availability

Not applicable.

## Abbreviations

2D: Two-dimensional
3D: Three-dimensional
3′UTR: Three prime untranslated region
AJ: Adherens junction
ARHGAP18: Rho GTPase activating protein 18
EMT: Epithelial-mesenchymal transition
ERK: Extracellular signal-regulated kinase
Ets-1: ETS Proto-oncogene 1
FAK: Focal adhesion kinase
FMNL3: Formin-like protein 3
GAP: GTPase activating protein
GEF: Guanine nucleotide exchange factor
Hop: Hsp90/Hsp70 organizing protein
Hsp: Heat shock protein
HUVEC: Human umbilical vein endothelial cells
IQGAP1: IQ motif-containing GTPase-activating protein 1
LIMK: Lin11/Isl1/Mec3 kinase
mDia1: Mammalian Diaphanous-related formin 1
MLC: Myosin light chain
MLCP: MLC phosphatase
MRK: MLK-related kinase
NADPH: Nicotinamide adenine dinucleotide phosphate
Notch1: Notch homolog 1, translocation-associated (*Drosophila*)
NOX4: NADPH oxidase 4
PBR: Polybasic region
PCACP: PEI-βCD@Ad-CDM-PEG
PDZ: PSD-95/Discs-large/ZO-1 homology
PI3K: Phosphoinositide 3-kinase
PKC: Protein kinase C
PTEN: Phosphatase and tensin homolog
ROCK: Rho-associated protein kinase
TEM4: Tumor endothelial marker 4
TJ: Tight junction
WASP: Wiskott-Aldrich syndrome protein
WAVE: WASP-family verprolin-homologous protein
